# The prevalence of anxiety disorders among young people in europe: A systematic review and meta-analysis

**DOI:** 10.1192/j.eurpsy.2021.1640

**Published:** 2021-08-13

**Authors:** R. Sacco, N. Camilleri, K. Umla-Runge

**Affiliations:** 1 Psychiatry, Cardiff University, Teesside University, Malta Mental Health Services, Attard, Malta; 2 Child And Young People’s Services, Malta Mental Health Services, Pieta, Malta; 3 Psychiatry, Cardiff University, Cardiff, United Kingdom

**Keywords:** Europe, Child, Anxiety, prevalence

## Abstract

**Introduction:**

This systematic review estimates the pooled prevalence (PP) of anxiety disorders (ADD) among 5-to-18-year-old YP living in Europe, based on prevalence rates established in the last five years (LFY).

**Objectives:**

Trends of prevalence rates across countries, gender and level of education were analysed. The random effects pooled prevalence rate (REPPR) for AD was calculated.

**Methods:**

A search strategy was conducted on three databases. Studies were also identified from reference lists and grey literature. Eligible studies were evaluated for reliability, validity, bias, and the REPPR for AD was calculated.

**Results:**

The European REPPR for AD is calculated at 7.9% (Figure 1). The REPPR for each anxiety disorder is shown in Figure 2.

**Figure:**
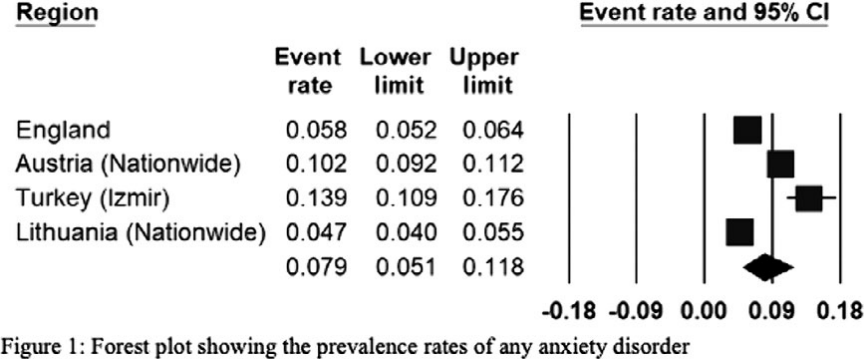

**Figure:**
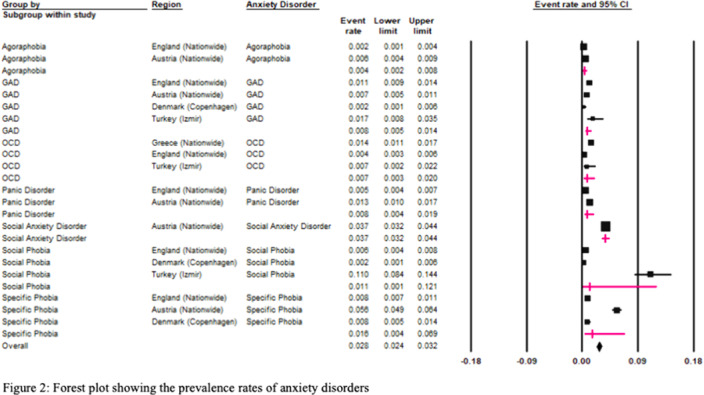

**Conclusions:**

Based on the results in this systematic review, AD are the most prevalent mental disorders among young people in Europe. Early diagnostic and intervention strategies for AD may improve the mental health and wellbeing among young people.

**Disclosure:**

No significant relationships.

